# Intrapulmonal dislocation of a totally implantable venous access device

**DOI:** 10.1186/1477-7819-3-19

**Published:** 2005-04-11

**Authors:** Thilo Hackert, Christin Tjaden, Angelika Kraft, Bernd Sido, Hendrik Dienemann, Markus W Buchler

**Affiliations:** 1 Dept. of Surgery, University of Heidelberg, Germany; 2 Department of Thoracic Surgery, University of Heidelberg, Germany

## Abstract

**Background:**

Totally implantable venous access devices are widely used for infusion of chemotherapy or parenteral nutrition. Device associated complications include technical operative problems, infections, paravasal infusions and catheter or punction chamber dislocation.

**Case presentation:**

We present the case of a 49-year-old patient with the rare complication of a intrapulmonal catheter dislocation of a totally implantable venous access system. Treosulfane for chemotherapy of metastatic breast cancer was infused via the catheter causing instant coughing and dyspnoea which lead to the diagnosis of catheter dislocation. The intrapulmonal part of the catheter was removed under thoracoscopic control without further complications.

**Conclusion:**

Intrapulmonal catheter dislocation is a rare complication of a totally implantable venous access device which can not be avoided by any prophylactic measures. Therefore, the infusion system should be tested before each use and each new symptom, even when not obviously related to the catheter should be carefully documented and evaluated by expert physicians to avoid severe catheter-associated complications.

## Background

Totally implantable venous access devices are widely used for infusion of chemotherapy or parenteral nutrition [1-4]. Implantation and use of these systems offer a high level of safety and convenience for patients and physicians. Device associated complications include technical operative problems, infections, paravasal infusions and catheter or punction chamber dislocation [1,2]. We present the case of a patient with the rare complication of an intrapulmonal catheter dislocation in a totally implantable venous access system.

## Case presentation

A 49-year-old female with metastatic breast cancer (supraclavicular lymph node metastases) presented with dyspnoea, intermittent coughing and general weakness. The patient had undergone chemotherapy with treosulfane via a totally implantable venous access port catheter the day before.

**Figure 1:**
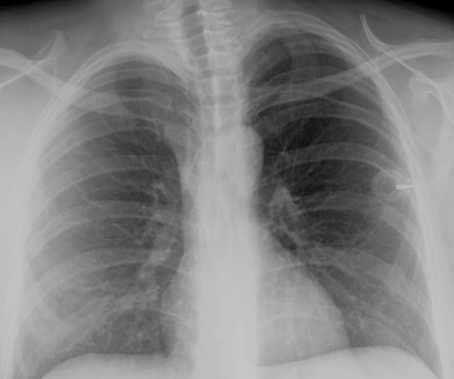
Postoperative x-ray after catheter implantation. The catheter tip is placed correctly in the superior caval vein.

The venous silicone catheter system (Fresenius Intraport, Fresenius Kabi, Bad Homburg, Germany) had been implanted 2 years before via the left cephalic vein by tangential incision of the vein after distal ligation without intra- or postoperative complications in our department. Correct placement had been documented by x-ray of the chest immediately after the implantation (figure 1). Following implantation, the catheter had been used for three months without any problems. Thereafter, the system was regularly flushed with heparin-saline solution. On presentation, injection into the port catheter was freely possible but caused instant reflectory coughing. Aspiration of blood via the port was not possible. Laboratory findings showed a mild leukocytosis of 11.0/nl and hypokalemia of 2.6 mmol/l.

**Figure 2:**
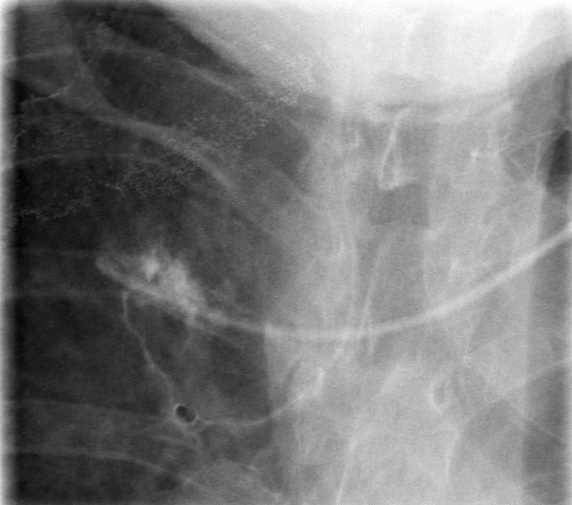
Injection of contrast medium via the port catheter. Paravasal and intrabronchial drainage of the applied contrast.

**Figure 3:**
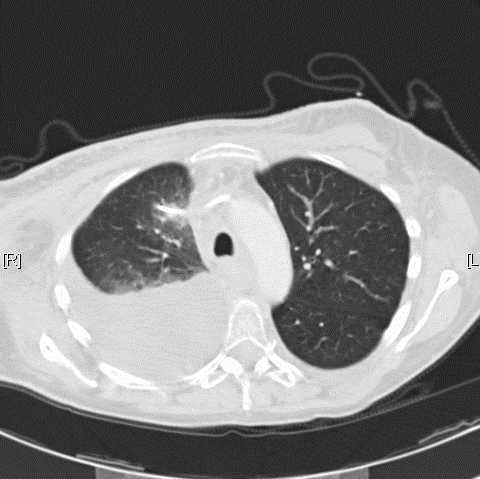
CT scan showing the catheter tip in the right upper lobe of the lung and a large dorsobasal pleural effusion.

Further radiological diagnostics including injection of contrast medium into the catheter documented a dislocation of the catheter tip into the upper lobe of the right lung with paravasation into the bronchial system (figure 2). In addition, thoracic computerised tomographic (CT) scan showed a large pleural effusion in the right pleural cavity (figure 3). There was no evidence for mediastinal or intrapulmonal tumour growth or lymph node metastases at the site of perforation. The patient was referred to the department of thoracic surgery for further therapy. The intrapulmonal tip of the port catheter was cut off and extracted thoracoscopically. The remaining catheter retracted into the superior caval vein lumen. As the patient had undergone mammarial gland ablation on both sides with consecutive radiation there was a severe dermatitis with ulcerations at the implantation site of the port catheter. Due to the high-risk of infection and wound healing complications and the limited life-expectancy of the patient, it was decided to leave the injection chamber and catheter remnant *in situ *without any further use for injections or infusions. The patient recovered from the intervention without complications.

## Discussion

Totally implantable venous access devices are broadly used for application of chemotherapy or intravenous nutrition, especially in patients with poor peripheral vein conditions [1-4]. Early, mainly surgical complications can occur, such as bleeding, pneumothorax, nerve lesions or catheter misplacement. Wound and catheter infection (4–5%), thrombosis (3–3.5%), catheter fracture or disconnection (0.5%) and secondary dislocation (1.5–2%) of the catheter are the most important long-term complications [1,2]. In the presented case, the catheter tip of the venous access device perforated the superior caval vein and dislocated into the upper lobe of the right lung. To our knowledge, this pulmonary complication has not been reported in the literature before. Common events of secondary dislocation include migration of the catheter tip into the internal jugular vein or the contralateral subclavian vein. A perforation of the caval vein and migration of the catheter into mediastinal structures or the pericardium has been reported [5-7]. The venous access device was implanted 2-years before and the correct placement of the catheter at the atrial-caval junction was documented by intra- and postoperative x-ray (figure 1). Moreover, the system worked properly since then, excluding a surgical problem in the presented case. The reason for the perforation remains obscure. CT scan showed no local inflammation, pathologic lymph nodes or tumour growth at the site of perforation. A spontaneous catheter perforation may be explained by a cranial dislocation of the catheter tip. A significant increase in catheter malfunctions has been reported by Petersen *et al*, [8] when catheters are placed primarily in a too high position in the caval vein. In the presented case this position might have resulted from a secondary dislocation. As the catheter was introduced via the left subclavian vein, its tip could get into a right angled position to the right lateral vessel wall of the caval vein. In this position a mechanical irritation leading to a chronic decubitus of the vessel wall with consequent perforation into the lung is possible. A similar event of an intrabronchial migration has been reported with a broken intraatrial pacing device [9]. However, these pacing catheter tips contain metallic material and therefore are more rigid than silicone port catheters, favouring a spontaneous perforation of these devices.

Another explanation for a chronic damage of the vessel wall might be endothelial cytotoxicity of the applied chemotherapy itself. In recent studies such an effect has been observed, especially when vinorelbin or 5-fluorouracil had been administered via a central venous catheter [10-12] leading to injury of the right phrenic nerve by direct cytotoxic effects. The authors of these studies postulated a damage of the endothelial barrier by the chemotherapeutic agent. In the presented case, first-line chemotherapy had included paclitaxel and epirubicin. Additionally, treosulfane had been administered immediately before the perforation became evident. None of these agents has yet been reported to cause endothelial damage as mentioned above. Therefore, a direct cytotoxic effect to the vessel wall seems rather unlikely.

Possible consecutive complications of the perforation itself include the risk of bleeding and air embolism as well as the paravasal application of fluids and especially aggressive chemotherapeutic agents via the dislocated catheter. There are case reports of accidental intrapericardial and intramediastinal applications of chemotherapeutic drugs as well as subcutaneous applications of chemotherapy due to wrong positioning of the puncture needle [5-7]. In most of the cases, patients did not suffer from adverse effects of these paravasats. In the presented case, treosulfane was administered intrabronchially, leading to coughing and dyspnoea, as well as a large pleural effusion which was absorbed without consecutive problems. Especially no interstitial pneumonia or evident tissue necrosis occurred.

Guidelines on how to avoid accidental paravasal infusion during the long-term use of port catheters include puncture and spilling of the catheter with saline solution, which is broadly accepted as a safety test before using the catheter. Blood aspiration before injection can be performed in addition. However, many catheters shows a "ventil" mechanism following long-term use. Therefore, blood aspiration may not be possible, although the catheter can still be used for infusion. A standard chest x-ray may be used to discover catheter dislocation, but does not reveal functional problems. The gold standard for diagnosis of catheter dislocation and function is the radiographic visualization with contrast medium application via the catheter. This is certainly no routine procedure prior to each application of chemotherapeutic drugs as it is associated with x-ray exposure of the patient, the risk of contrast-medium related complications, requires radiological facilities and high costs. Therefore, the only recommendation is to puncture and use port catheters with the highest accuracy. Each new symptom, even when not obviously related to the catheter, e.g. coughing following infusion, should be carefully documented and evaluated by expert physicians to avoid severe catheter-associated complications.

## Conclusion

Intrapulmonal dislocation of the catheter tip is a rare complication of a totally implantable venous access device. However, it can cause severe complications and may be difficult to recognize due to unspecific symptoms.

## Authors' contributions

TH, CT review of literature and manuscript preparation

AK, HD surgical management

BS, MWB review of manuscript

All authors have read and approved the manuscript in the presented form.

## Competing Interests

The author(s) declare that they have no competing interests.
